# Elevated SGK1 predicts resistance of breast cancer cells to Akt inhibitors

**DOI:** 10.1042/BJ20130342

**Published:** 2013-05-31

**Authors:** Eeva M. Sommer, Hannah Dry, Darren Cross, Sylvie Guichard, Barry R. Davies, Dario R. Alessi

**Affiliations:** *MRC Protein Phosphorylation and Ubiquitylation Unit, College of Life Sciences, University of Dundee, Dundee DD1 5EH, U.K.; †Oncology iMED, AstraZeneca, Alderley Park, Cheshire SK10 4TG, U.K.

**Keywords:** mammalian target of rapamycin (mTOR), N-Myc downstream-regulated gene 1 (NDRG1), protein kinase inhibitor, phosphoinositide 3-kinase (PI3K), signal transduction inhibitor, FBS, fetal bovine serum, FOXO, forkhead box O, GI_50_, growth inhibition by 50%, GSK3, glycogen synthase kinase 3, HEK, human embryonic kidney, HRP, horseradish peroxidase, mTOR, mammalian target of rapamycin, mTORC, mTOR complex, MTS, 3-(4,5-dimethylthiazol-2-yl)-5-(3-carboxymethoxyphenyl)-2-(4-sulfophenyl)-2*H*-tetrazolium, N-Myc, neuroblastoma-derived Myc, NDRG1, N-Myc downstream-regulated gene 1, PDK1, phosphoinositide-dependent kinase 1, PH, pleckstrin homology, PI3K, phosphoinositide 3-kinase, PRAS40, proline-rich Akt substrate of 40 kDa, PTEN, phosphatase and tensin homologue deleted on chromosome 10, qRT-PCR, quantitative reverse transcription–PCR, SGK, serum- and glucocorticoid-regulated kinase, shRNA, short hairpin RNA, TBST, Tris-buffered saline-Tween

## Abstract

The majority of human cancers harbour mutations promoting activation of the Akt protein kinase, and Akt inhibitors are being evaluated in clinical trials. An important question concerns the understanding of the innate mechanisms that confer resistance of tumour cells to Akt inhibitors. SGK (serum- and glucocorticoid-regulated kinase) is closely related to Akt and controlled by identical upstream regulators {PI3K (phosphoinositide 3-kinase), PDK1 (phosphoinositide-dependent kinase 1) and mTORC2 [mTOR (mammalian target of rapamycin) complex 2]}. Mutations that trigger activation of Akt would also stimulate SGK. Moreover, Akt and SGK possess analogous substrate specificities and are likely to phosphorylate overlapping substrates to promote proliferation. To investigate whether cancers possessing high SGK activity could possess innate resistance to Akt-specific inhibitors (that do not target SGK), we analysed SGK levels and sensitivity of a panel of breast cancer cells towards two distinct Akt inhibitors currently in clinical trials (AZD5363 and MK-2206). This revealed a number of Akt-inhibitor-resistant lines displaying markedly elevated SGK1 that also exhibited significant phosphorylation of the SGK1 substrate NDRG1 [N-Myc (neuroblastoma-derived Myc) downstream-regulated gene 1]. In contrast, most Akt-inhibitor-sensitive cell lines displayed low/undetectable levels of SGK1. Intriguingly, despite low SGK1 levels, several Akt-inhibitor-sensitive cells showed marked NDRG1 phosphorylation that was, unlike in the resistant cells, suppressed by Akt inhibitors. SGK1 knockdown markedly reduced proliferation of Akt-inhibitor-resistant, but not -sensitive, cells. Furthermore, treatment of Akt-inhibitor-resistant cells with an mTOR inhibitor suppressed proliferation and led to inhibition of SGK1. The results of the present study suggest that monitoring SGK1 levels as well as responses of NDRG1 phosphorylation to Akt inhibitor administration could have a use in predicting the sensitivity of tumours to compounds that target Akt. Our findings highlight the therapeutic potential that SGK inhibitors or dual Akt/SGK inhibitors might have for treatment of cancers displaying elevated SGK activity.

## INTRODUCTION

Over 70% of breast cancers possess mutations that trigger activation of the PI3K (phosphoinositide 3-kinase) signalling pathway [1]. These include mutations that induce overexpression of receptor tyrosine kinases [e.g. HER-2 (human epidermal growth factor receptor 2)], loss of the tumour suppressor 3-phosphoinositide phosphatase PTEN (phosphatase and tensin homologue deleted on chromosome 10) or constitutively activate PI3K [2]. Given the pivotal role of PI3K signalling in controlling cell growth, survival and proliferation, key components of this pathway, PI3K, mTOR (mammalian target of rapamycin) and Akt, have emerged as promising targets for cancer drug discovery [2,3]. Much research has focused on the role of Akt isoforms in driving proliferation of tumour cells. Akt is stimulated following activation of PI3K by growth factor receptors or Ras proteins at the plasma membrane. PI3K phosphorylates the membrane phospholipid PtdIns(4,5)*P*_2_ to yield PtdIns(3,4,5)*P*_3_ [4]. This allows for the colocalization of Akt and PDK1 (phosphoinositide-dependent kinase 1) at the plasma membrane via their PtdIns(3,4,5)*P*_3_-binding PH (pleckstrin homology) domains and for efficient activation of Akt by PDK1 via phosphorylation of Akt at Thr^308^ [5]. The activity of Akt is further positively controlled by mTORC (mTOR complex) 2-mediated phosphorylation of Akt at Ser^473^ [6]. Phosphorylation of Ser^473^ also promotes the phosphorylation of Akt at Thr^308^ by PDK1 [7]. Akt regulates cell survival by phosphorylating multiple targets including FOXO (forkhead box O) transcription factors and GSK3 (glycogen synthase kinase 3). Furthermore, by phosphorylating TSC2 (tuberous sclerosis complex 2) and PRAS40 (proline-rich Akt substrate of 40 kDa), Akt promotes activation of mTORC1 that plays a vital role in orchestrating proliferation responses [8].

Although most work has focused on Akt as being the major mediator of cell proliferation induced by activation of PI3K, a closely related enzyme termed SGK (serum- and glucocorticoid-regulated kinase), of which three isoforms exist, has by comparison received little attention. Although SGK isoforms lack an N-terminal PtdIns(3,4,5)*P*_3_-binding PH domain, the kinase domains of Akt and SGKs share approximately 50% identity [9–11]. Furthermore, PI3K activation triggers the stimulation of SGK via a similar mechanism to Akt. PI3K activation induces mTORC2 phosphorylation of the hydrophobic motif of SGK isoforms thereby promoting phosphorylation of the T-loop residue by PDK1, which activates SGKs [12–14]. Although there are subtle differences in the optimal substrate specificity requirements of SGK and Akt kinases [15], both enzymes phosphorylate substrates within a similar Arg-Xaa-Arg-Xaa-Xaa-Ser/Thr (where Xaa is any amino acid) consensus sequence [12,16]. Indeed, many Akt substrates that have been evaluated, such as FOXO transcription factors [17] or GSK3 [12], are similarly phosphorylated *in vitro* by SGK isoforms. Consequently it is likely that Akt and SGK isoforms could phosphorylate an overlapping set of substrates and hence possess similar functions such as promoting proliferation and survival of cancer cells.

There are currently 217 clinical trials listed on the NIH clinical trials website that have been initiated or planned to evaluate the therapeutic efficacy of Akt inhibitors for the treatment of cancer (http://www.clinicaltrials.gov/). The first phase one report of a clinical trial with the highly specific non-ATP competitive allosteric Akt inhibitor termed MK-2206 has been reported recently [18]. The ability to predict which tumours will be most responsive to Akt inhibitors is an important question and of relevance to Akt inhibitor clinical trials. Owing to the similarity of SGK and Akt isoforms and the potential that these enzymes possess analogous functions, we investigated whether tumour cells displaying high levels of SGK activity would be more resistant to Akt inhibitors than tumours lacking SGK. Expression of SGK isoforms is much more variable between cells and tissues than Akt [19,20], suggesting that only a subset of tumour cells would possess elevated SGK activity. We identified a number of Akt-inhibitor-resistant breast cancer cells that possess elevated levels of SGK1 and present evidence that SGK1 represents a major driver of proliferation in these cells. In contrast, all Akt-inhibitor-sensitive cells analysed displayed low or undetectable levels of SGK1 protein. The findings from the present study indicate that monitoring SGK1 levels as well the affect that administration of Akt inhibitors has on NDRG1 [N-Myc (neuroblastoma-derived Myc) downstream-regulated gene 1] phosphorylation could have utility in predicting the sensitivity of tumours to Akt inhibitors. The results also suggest that SGK inhibitors or dual Akt and SGK inhibitors might have utility for treating cancers displaying elevated SGK activity.

## MATERIALS AND METHODS

### Materials

MK-2206 was synthesized by Dr Natalia Shpiro at the University of Dundee, AZD5363 was generated as described previously [21] and AZD8055 was from Axon Medchem. DMSO and Tween 20 were from Sigma. CellTiter 96® AQueous One Solution Cell Proliferation Assay {MTS [3-(4,5-dimethylthiazol-2-yl)-5-(3-carboxymethoxyphenyl)-2-(4-sulfophenyl)-2*H*-tetrazolium]} was from Promega. Enhanced chemiluminescence reagent was from GE Healthcare. IGF1 (insulin-like growth factor 1) was from Cell Signaling Technology.

### Antibodies

The following antibodies were raised by the Division of Signal Transduction Therapy (DSTT) at the University of Dundee in sheep and affinity-purified against the indicated antigens: anti-Akt1 (S695B, third bleed; raised against residues 466–480 of human Akt1: RPHFPQFSYSASGTA), anti-PRAS40 (S115B, first bleed; raised against residues 238–256 of human PRAS40: DLPRPRLNTSDFQKLKRKY), anti-(phospho-PRAS40 Thr^246^) (S114B, second bleed; raised against residues 240–251 of human PRAS40: CRPRLNTpSDFQK), anti-NDRG1 (S276B third bleed; raised against full-length human NDRG1) and anti-SGK3 (S037D third bleed; raised against human SGK3 PX (Phox homology) domain comprising residues 1–130 of SGK3). Anti-(phospho-Akt Ser^473^) (#9271), anti-(phospho-Akt Thr^308^) (#4056), anti-(phospho-S6 ribosomal protein Ser^235^/Ser^236^) (#4856), anti-(total S6 ribosomal protein) (#2217), anti-PTEN (#9559) and anti-phospho-NDRG1 Thr^346^ (#5482) antibodies were purchased from Cell Signaling Technology. For immunoblotting of the phosphorylated T-loop of SGK1, we employed the pan-PDK1 site antibody from Cell Signaling Technology (#9379) as previously described [21a]. We have confirmed that this pan-PDK1 site antibody efficiently recognizes the phosphorylated T-loop of SGK1 (Supplementary Figure S1 at http://www.biochemj.org/bj/452/bj4520499add.htm). Anti-(phospho-SGK1 Ser^422^) (#sc16745) antibody was from Santa Cruz Biotechnology and total SGK (#5188) antibody was from Sigma. Secondary antibodies coupled to HRP (horseradish peroxidase) were obtained from Thermo Scientific.

### General methods

Restriction enzyme digests, DNA ligations and other recombinant DNA procedures were performed using standard protocols. DNA constructs used for transfection were purified from *Escherichia coli* DH5α cells using a Qiagen plasmid Maxi prep kit according to the manufacturer's protocol. All DNA constructs were verified by DNA sequencing, which was performed by DNA Sequencing and Services (MRCPPU, College of Life Sciences, University of Dundee, Scotland; http://www.dnaseq.co.uk) using Applied Biosystems Big-Dye Ver 3.1 chemistry on an Applied Biosystems model 3730 automated capillary DNA sequencer.

### Buffers

The following buffers were used: lysis buffer (50 mM Tris/HCl, pH 7.5, 1% Triton X-100, 1 mM EGTA, 1 mM EDTA, 150 mM NaCl, 0.27 M sucrose, 50 mM sodium fluoride, 10 mM sodium 2-glycerophosphate, 5 mM sodium pyrophosphate, 1 mM sodium orthovanadate, 1 mM benzamidine, 1 mM PMSF and 0.1% 2-mercaptoethanol), TBST (Tris-buffered saline-Tween) (50 mM Tris/HCl, pH 7.5, 0.15 M NaCl and 0.1% Tween 20) and sample buffer [50 mM Tris/HCl, pH 6.8, 6.5% (v/v) glycerol, 1% (w/v) SDS and 1% (v/v) 2-mercaptoethanol].

### Immunoblotting

Total cell lysate samples (10–20 μg) were heated at 95°C for 5 min in sample buffer, subjected to SDS/PAGE (10%) and transferred on to nitrocellulose membranes. Membranes were blocked for 1 h in TBST containing 5% (w/v) non-fat dried skimmed milk powder. Membranes were probed with the indicated antibodies in TBST containing 5% (w/v) non-fat dried skimmed milk powder or BSA for 16 h at 4°C. Detection was performed using HRP-conjugated secondary antibodies and enhanced chemiluminescence reagent.

### Cell culture

Cell lines were sourced as described previously [21] and were cultured in RPMI 1640 medium supplemented with 10% (v/v) FBS (fetal bovine serum), 2 mM L-glutamine, 100 units/ml penicillin and 0.1 mg/ml streptomycin at 37°C in 5% CO_2_. Cells were treated with inhibitors diluted in DMSO as described in the Figure legends. Prior to lysis, cells were rinsed with PBS and then lysed on ice. Lysates were snap-frozen in liquid nitrogen, centrifuged at 18000 ***g*** for 15 min at 4°C and supernatants were stored at −80°C. For transient transfections of HEK (human embryonic kidney)-293 cells, cells cultured on 10-cm-diameter dishes were transfected with 5 μg of the indicated plasmids using polyethylenimine.

### Cell growth and invasion assays

Cells (3000–4000 per well) were seeded into the inner 60 wells of 96-well plates in triplicate and allowed to attach overnight. For inhibitor treatments, cells were treated with 10 nM–10 μM MK-2206, AZD5363 or AZD8055 diluted in DMSO. At 72 h later cell viability was determined using CellTiter 96® AQueous One Solution Cell Proliferation Assay (MTS) according to the manufacturer's instructions. Results were plotted with a best-fit sigmoidal variable slope dose–response curve and GI_50_ (growth inhibition by 50%) values were calculated using GraphPad Prism 5.0. Breast cell line panel screening for AZD5363 was carried out as described previously [21].

The capacity for proliferation following SGK1 knockdown was determined by seeding 2000 cells/well into the inner 60 wells of 96-well plates 48 h post-transduction with lentiviral shRNAs (short hairpin RNAs). The MTS assay was then performed 24, 48, 72, 96, 120 and 144 h post-seeding. Results are presented as the fold change in absorbance over the 5-day period relative to the assay start point (24 h post-seeding or day 0). The cells were assayed in triplicate.

The ability of BT-549 cells to invade was tested in a growth-factor-reduced Matrigel™ invasion chamber (BD Biosciences #354483) according to the manufacturer's instructions. Briefly, cells were serum-deprived for 2 h, detached with a cell-dissociation buffer (Gibco) and 2.5×10^5^ cells suspended in RPMI 1640 medium containing 1% (w/v) BSA were added to the upper chambers in triplicate and chemoattractant [RPMI 1640 medium containing 10% (v/v) FBS] was added to the lower wells. The chambers were kept at 37°C in 5% CO_2_ for 20 h. Cells that did not invade were removed from the upper face of the filters and cells that had migrated to the lower face of the filters were fixed and stained with Reastain Quick-Diff kit (Reagena) and images (×10 magnification) were captured. For cell invasion assays, statistical significance was assessed by one-way ANOVA followed by Tukey's multiple comparison test using GraphPad Prism 5.0.

### shRNA-mediated SGK1 knockdown using a lentiviral delivery system

To knock down SGK1 we used the MISSION™ shRNA system obtained from Sigma–Aldrich. Five separate pLK0.1 lentiviral-plasmid-based shRNA constructs specific for human SGK1 (#SHCLNG-NM_005627) were used along with scrambled shRNA cloned into the same pLK0.1 vector. shRNA sequences are shown in Supplementary Table S1 (at http://www.biochemj.org/bj/452/bj4520499add.htm).

To generate lentiviral particles, HEK-293T cells grown on 10-cm-diameter dishes were transfected with 3 μg of the shRNA-encoding plasmid (pLK0.1), 3 μg of pCMV delta R8.2 (packaging plasmid) and 3 μg of pCMV-VSV-G (envelope plasmid) using polyethylenimine. At 72 h post-transfection, virus-containing medium was collected and filtered with a 0.45 μm filter. A total of 2 ml of viral supernatant was used to infect cells cultured in six-well plates in the presence of 8 μg/ml polybrene (sc-134220, Santa Cruz Biotechnology). After 24 h, virus-containing medium was replaced with fresh medium and, after 24 h, cells were seeded for experiments. Protein knock down was assayed by immunoblotting 72 h post-infection. No antibiotic selection was used as in initial optimization experiments infection efficiency was found to be near 100%.

### Rescue of shRNA-mediated SGK1 knockdown

Wild-type and catalytically inactive (K127A) SGK1 lacking the N-terminal 1–60 amino acids (ΔN-SGK1) described previously [22] were subcloned into pBABE-puro-HA retroviral vector (Cell Biolabs). To generate retroviral particles, HEK-293T cells grown on 10-cm-diameter dishes were transfected with 6 μg of pBABE construct, 3.8 μg of pCMV-Gag-Pol and 2.2 μg of pCMV-VSVG using Lipofectamine™ 2000 (Invitrogen) according to the manufacturer's instructions. At 72 h post-transfection, virus-containing medium was collected and filtered with a 0.45 μm filter. A total of 2 ml of viral supernatant was used to infect BT-549 cells cultured in six-well plates in the presence of 8 μg/ml polybrene (sc-134220, Santa Cruz Biotechnology). After 24 h of incubation with virus-containing medium, the medium was replaced with fresh medium and, after 24 h, transduced cells were selected in the presence of 2 μg/ml puromycin. For rescue experiments, BT-549 cells stably expressing wild-type or kinase-inactive SGK1 were infected with SGK1 shRNA SGK1 #D that targets the 3′-UTR (untranslated region) or scrambled shRNA. Rescue experiments were performed in the absence of puromycin.

### RNA isolation, cDNA preparation and analysis of transcripts by qRT-PCR (quantitative reverse transcription–PCR)

Total RNA was isolated from cells using the RNeasy kit (Qiagen). cDNA was prepared from 1 μg of RNA using the i-Script Kit (Bio-Rad Laboratories). qRT-PCR was performed in 96-well plate format using iQ5™ Real Time PCR detection system (Bio-Rad Laboratories), where each 20 μl reaction included 1% cDNA preparation, 0.5 μM primers and 10 μl SYBR Green (Bio-Rad Laboratories). The primer sequences used are shown in Supplementary Table S2 (at http://www.biochemj.org/bj/452/bj4520499add.htm). The data presented are representative of two independent biological repeats each assayed in duplicate and are relative expression levels. Data were normalized to an internal standard gene 18S and are presented as relative levels in comparison with the SGK isoform mRNA levels in HEK-293 cells.

Whole transcriptome (Affymetrix U133) analysis was carried out and analysed as described previously [23]. Data was collapsed gene centrically and scaled relative to the expression range across a panel of 500 cell lines (0=minimum and 1=maximum).

## RESULTS

### Identification of Akt-inhibitor-sensitive and -resistant breast cancer cell lines

**Figure 1:**
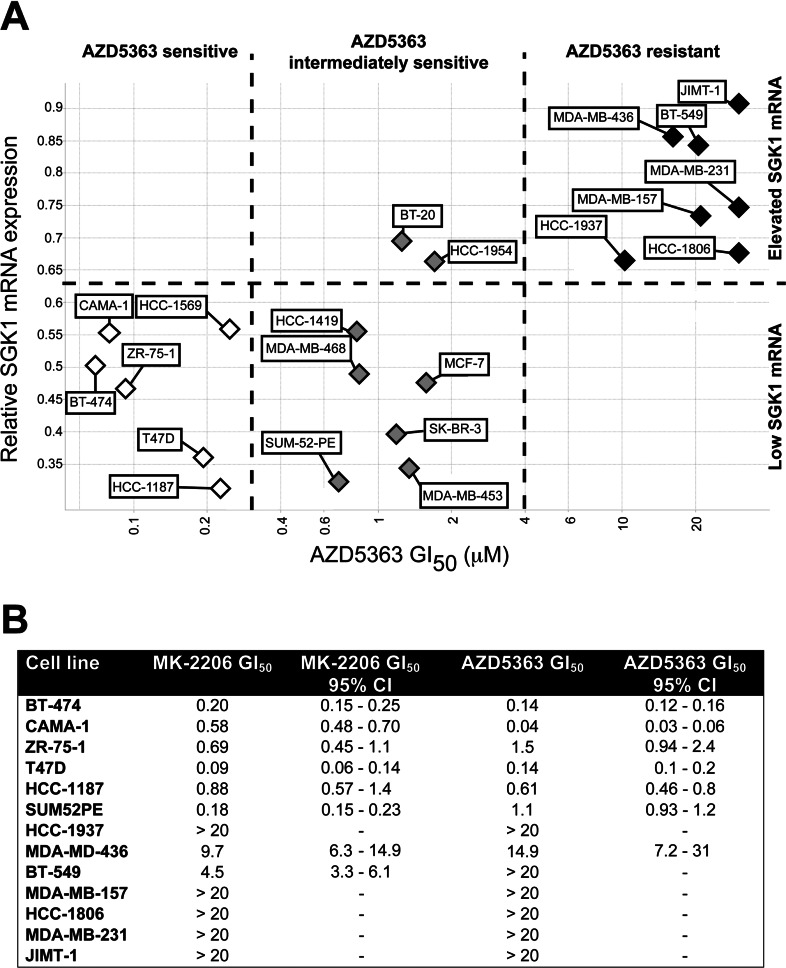
Sensitivity of breast cancer cell lines to Akt inhibition correlates with *SGK1* mRNA levels (**A**) A panel of 21 breast cancer cell lines were screened in a standard 72 h MTS cell proliferation assay. Cell lines with a GI_50_ value of <3 μM were classified as sensitive (open diamonds), cell lines with a GI_50_ value of >3 μM were classified as resistant (closed diamonds) and cell lines with a sensitivity between the two were classified as intermediately sensitive (grey diamonds). *SGK1* gene expression from whole transcriptome analysis (Affymetrix U133) was scaled relative to the expression range across a panel of 500 cell lines where 0=minimum and 1=maximum. (**B**) The indicated cells were exposed to increasing concentrations (0–10 μM) of MK-2206 or AZD5363 for 72 h and cell viability was determined using the MTS assay. Data were fitted to sigmoidal dose–response curves and GI_50_ values and 95% confidence intervals (CI) were calculated using GraphPad Prism 5.0. Similar results were observed in two independent experiments. Where GI_50_ values exceeded 20 μM, data were found to fit poorly to sigmoidal dose–response curves, and therefore no exact GI_50_ values or 95% CIs are stated for these samples.

To establish whether there was a link between resistance to Akt inhibitors and SGK1 expression, we first compared the GI_50_ values mediated by the Akt inhibitor AZD5363 (that does not significantly inhibit SGK1) [21] with relative *SGK1* mRNA levels in 21 breast cancer cell lines (Figure 1A). This strikingly revealed that there was a group of highly AZD5363-sensitive cell lines with low *SGK1* mRNA expression (BT-474, CAMA-1, ZR-75-1, T47D, HCC-1187 and HCC-1569) and a group of resistant cell lines with high *SGK1* mRNA expression (HCC-1937, MDA-MB-436, BT-549, MDA-MB-157, MDA-MB-231, HCC-1806 and JIMT-1) (Figure 1A). There were eight cell lines that displayed intermediate sensitivity towards AZD5363, six having low *SGK1* mRNA levels (SUM-52-PE, MDA-MB-453, SK-BR-3, MCF-7, MDA-MB-468 and HCC-1419) and two with elevated *SGK1* mRNA levels (BT-20 and HCC-1954) (Figure 1A). We chose five sensitive (BT-474, CAMA-1, ZR-75-1, T47D and HCC-1187), one intermediate (SUM-52-PE) and the seven resistant (HCC-1937, MDA-MB-436, BT-549, MDA-MB-157, MDA-MB-231, HCC-1806 and JIMT-1) cell lines for further analysis. The known mutations in each of these cells are listed in Supplementary Table S3 (at http://www.biochemj.org/bj/452/bj4520499add.htm). We confirmed that many of these cells also displayed similar sensitivity towards the structurally distinct allosteric Akt inhibitor MK-2206 (that also does not significantly inhibit SGK1 activity) [24] (Figure 1B).

### Analysis of SGK and Akt in Akt-inhibitor-sensitive and -resistant cells

**Figure 2:**
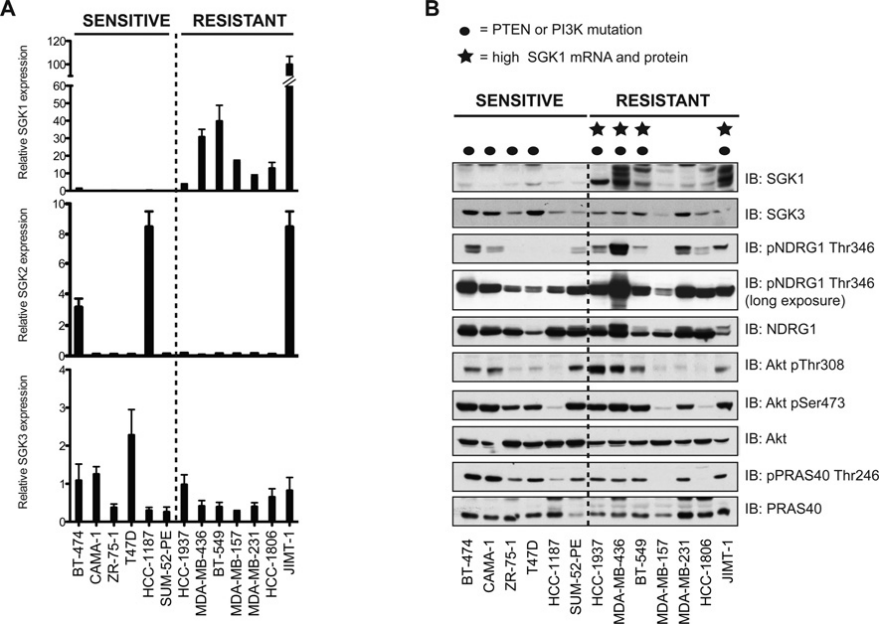
Identification of Akt-inhibitor-resistant cell lines that possess elevated SGK1 protein and mRNA levels (**A**) Total mRNA was isolated from cells and SGK isoform mRNA levels were determined by qRT-PCR. The data were normalized to an internal 18S control and are presented as relative levels in comparison with control cells (HEK-293). Results represent the means±S.E.M. of two independent samples each assayed in duplicate. (**B**) Total protein was isolated from cells and analysed by immunoblotting (IB) with the indicated antibodies. Similar results were observed in two independent experiments. p, phospho-.

Using qRT-PCR we studied the relative mRNA expression of all three SGK isoforms in the Akt-inhibitor-sensitive and -resistant cell lines (Figure 2A). This revealed that all seven Akt-inhibitor-resistant cell lines selected displayed significantly higher levels of *SGK1* mRNA than the six sensitive cell lines. In contrast, levels of *SGK2* mRNA were variable between sensitive and resistant cells. We found that *SGK3* mRNA levels were present at more similar levels across the resistant and sensitive cell lines (Figure 2A).

We next undertook an immunoblot analysis of the relative expression levels of SGK1 and SGK3 as well as phosphorylation levels of NDRG1 at Thr^346^, which is an SGK phosphorylation site [25,26]. This revealed that four of the Akt-inhibitor-resistant cell lines (HCC-1937, MDA-MB-436, BT-549 and JIMT-1) possessed a readily detectable elevated SGK1 protein expression and also displayed high levels of NDRG1 phosphorylation. All of these cell lines possess mutations that would be expected to activate PI3K. HCC-1937, MDA-MB-436 and BT-549 cells were null for PTEN protein expression (Supplementary Figure S2 and Supplementary Table S3 at http://www.biochemj.org/bj/452/bj4520499add.htm) whereas JIMT-1 cells have an activating mutation in the catalytic subunit of PI3K (C420R) [27]. Two of the remaining Akt-inhibitor-resistant cell lines (HCC-1806 and MDA-MB-231), although not displaying obvious elevation of SGK1 protein, nevertheless exhibited significant phosphorylation of NDRG1 (Figure 2B). One of the seven Akt-inhibitor-resistant cell lines investigated (MDA-MB-157) showed no detectable SGK1 protein and low levels of NDRG1 phosphorylation (Figure 2B) suggesting that SGK signalling is not activated in these cells.

We also monitored Akt expression and activity by assessing Thr^308^ and Ser^473^ phosphorylation as well as phosphorylation of the Akt substrate PRAS40. We found that all of the Akt-inhibitor-sensitive cells and five out of the seven resistant cells (HCC-1937, MDA-MB-436, BT-459, HCC-1806 and JIMT-1) displayed significant Akt Thr^308^/Ser^473^ and PRAS40 Thr^246^ phosphorylation, confirming that the Akt signalling pathway is active in these cells. In contrast, resistant MDA-MB-157 and HCC-1806 cells had very low levels of Akt Thr^308^/Ser^473^ and undetectable PRAS40 Thr^246^ phosphorylation (Figure 2B).

### Knockdown of SGK1 impairs proliferation of Akt-inhibitor-resistant cells

**Figure 3:**
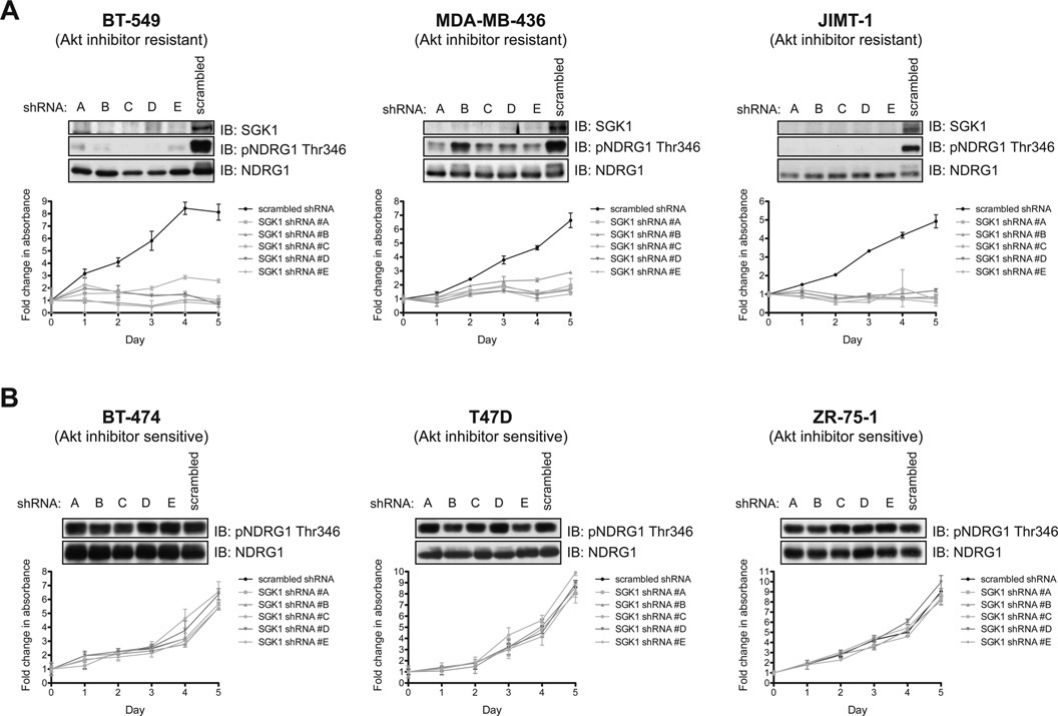
Knockdown of SGK1 impairs proliferation of Akt-inhibitor-resistant cells but not -sensitive cells The indicated Akt-inhibitor-resistant (**A**) and sensitive (**B**) cells were transduced with lentiviral SGK1 and scrambled shRNAs. For experiments shown in the upper panels, cells were lysed 72 h post-infection and the lysates were analysed with the antibodies indicated by immunoblotting (IB). For experiments shown in the lower panel, equal numbers of cells were seeded onto 96-well plates 48 h post-infection and allowed to adhere overnight. Cell proliferation was then determined over a 5 day period by carrying out the MTS assay at 24-h intervals. Each data point is the average MTS assay of samples assayed in triplicate±S.D. The data are presented as fold change relative to day 0 values (day 0 equals 24 h post-seeding). Similar results were observed in at least two independent experiments. p, phospho-.

SGK1 has a short half-life, making it straightforward to knockdown SGK1 protein expression using RNA interference [28]. Employing a lentiviral shRNA approach we identified five independent shRNAs that reduced the expression of SGK1 protein to near undetectable levels in the Akt-inhibitor-resistant cell lines displaying high levels of SGK1 protein (BT-549, MDA-MB-436 and JIMT-1, Figure 3A, or HCC-1937, Supplementary Figure S3 at http://www.biochemj.org/bj/452/bj4520499add.htm). Consistent with the efficient knockdown of SGK1 protein, all shRNA probes markedly reduced NDRG1 phosphorylation in the resistant cell lines (Figure 3A and Supplementary Figure S3). Strikingly, knockdown of SGK1 protein markedly impaired proliferation of all Akt-inhibitor-resistant cell lines examined (Figure 3A and Supplementary Figure S3). In contrast, treatment of Akt-inhibitor-sensitive cells (BT-474, T47D and ZR-75-1) with SGK1 shRNA lentivirus had no affect on NDRG1 phosphorylation or proliferation (Figure 3B).

**Figure 4:**
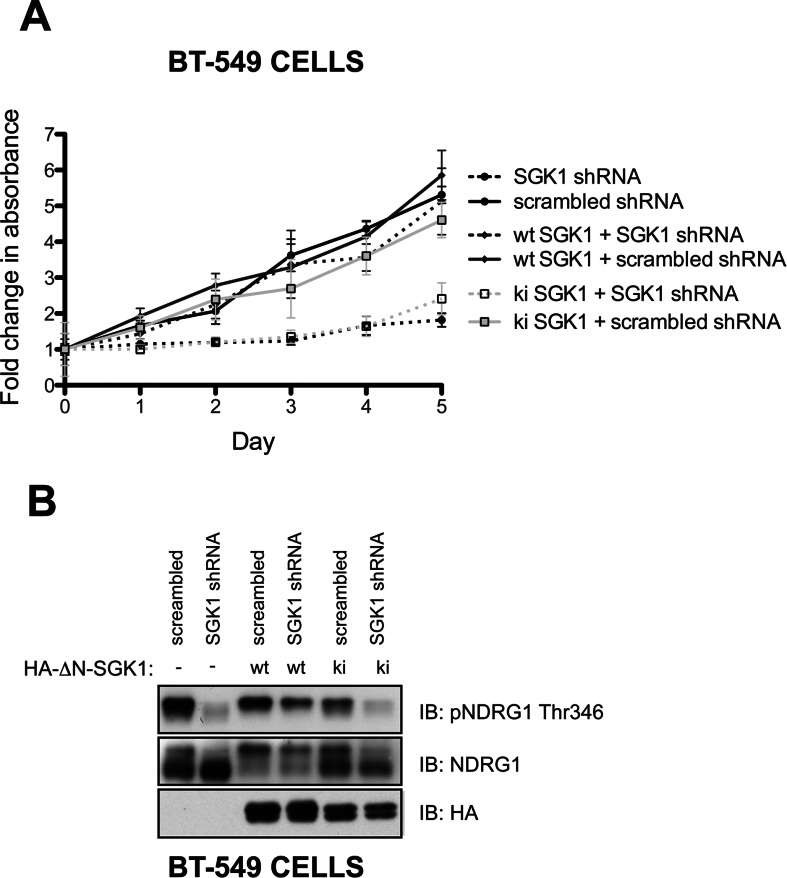
SGK1 knockdown-induced proliferation impairment can be rescued by ectopic expression of wild-type SGK1 BT-549 cells stably expressing retroviral HA (haemagglutinin)–ΔN-SGK1 wild-type (wt) or kinase-inactive (ki) constructs were transduced with lentiviral SGK1 shRNA #D or scrambled shRNA. This construct of SGK1 lacks the N-terminal 1–60 residues that contain a motif that targets SGK1 for proteasomal degradation and therefore enables SGK1 to be expressed at a detectable level [41]. (**A**) Equal numbers of cells were seeded 48 h post-infection on to 96-well plates and allowed to adhere overnight. Cell proliferation was then determined by carrying out the MTS assay over a 5 day period with cells assayed at 24 h intervals. Each data point is the average MTS assay of samples assayed in triplicate±S.D. The data are presented as fold change relative to day 0 values (day 0 equals 24 h post-seeding). (**B**) Cells were lysed 72 h post-infection with shRNAs and analysed by immunoblotting (IB) with the indicated antibodies. Similar results were observed in at least two independent experiments. p, phospho-.

To confirm that inhibition of proliferation following knockdown of SGK1 in Akt-inhibitor-resistant BT-549 cells was indeed mediated by a reduction in SGK1 activity, we undertook a rescue experiment. Endogenous SGK1 expression was knocked down in BT-549 cells stably overexpressing wild-type or kinase-inactive SGK1. This experiment revealed that, in BT-549 cells lacking endogenous SGK1, growth and NDRG1 phosphorylation could be rescued by overexpression of wild-type, but not kinase-inactive SGK1 (Figure 4).

### Knockdown of SGK1 reduces the invasive ability of BT-549 cells

**Figure 5:**
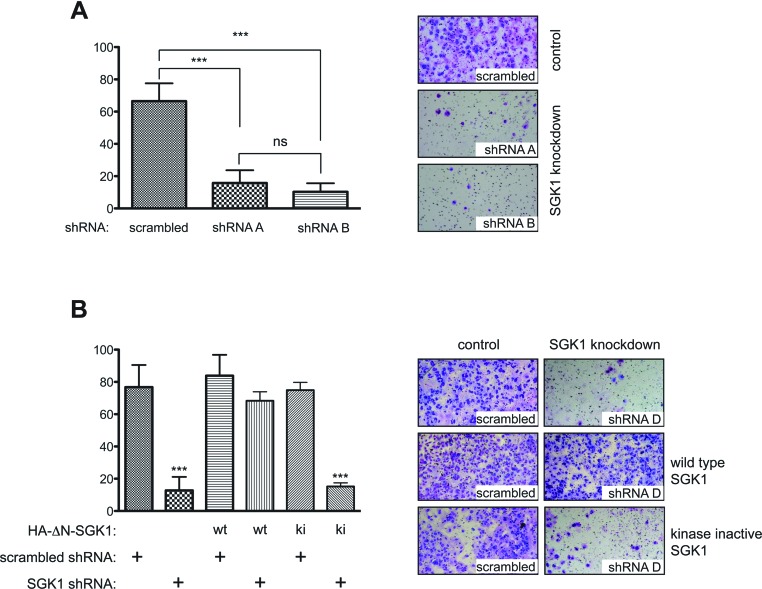
Knockdown of SGK1 reduces the invasive ability of BT-549 cells (**A**) BT-549 cells were transduced with lentiviral SGK1 or scrambled shRNAs. Cells were plated for a transwell invasion assay 48 h post-infection in triplicate using 10% FBS as a chemoattractant. Cells that had invaded through to the lower face of the filters were fixed and photographed (×10 magnification). (**B**) BT-549 cells stably expressing retroviral HA (haemagglutinin)–ΔN-SGK1 wild-type (wt) or kinase-inactive (ki) constructs were transduced with lentiviral SGK1 shRNA or scrambled shRNA and 48 h post-infection a transwell invasion assay was performed as above. The cells that migrated were counted and the data are presented as the mean number of migrated cells±S.D. Statistical significance was assessed by ANOVA followed by Tukey's multiple comparison test using GraphPad Prism 5.0. ***P<0.001 compared with the scrambled control; ns, not significant. Similar results were observed in at least two independent experiments.

Because SGK1 has previously been reported to be important for the ability of various cell types to migrate [29,30], we tested whether SGK1 knockdown impaired the ability of the highly invasive BT-549 cells to invade into a three-dimensional Matrigel matrix in a transwell invasion assay. We found that knockdown of SGK1 by two independent shRNAs markedly affected the invasiveness of BT-549 cells in this assay (Figure 5A). To confirm that the impaired invasion was due to SGK1 knockdown, we performed a rescue experiment. Overexpression of wild-type, but not kinase-inactive SGK1, was found to restore invasiveness of SGK1 shRNA-infected cells back to control levels (Figure 5B).

### Evidence that Akt mediates phosphorylation of NDRG1 in Akt-inhibitor-sensitive cells

**Figure 6:**
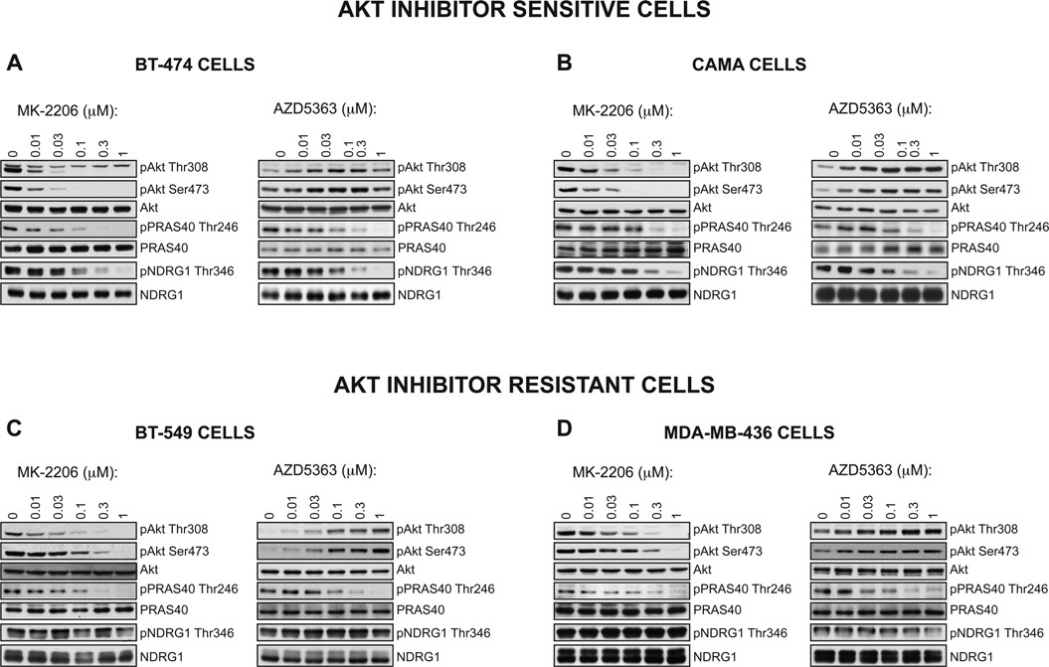
Evidence that Akt mediates phosphorylation of NDRG1 in Akt-inhibitor-sensitive cells The indicated Akt-inhibitor-sensitive (**A** and **B**) and resistant (**C** and **D**) cells were treated with the indicated doses of the MK-2206 or AZD5363 Akt inhibitors for 1 h. Cells were lysed and analysed by immunoblotting with the indicated antibodies. Similar results were observed in two independent experiments.

Interestingly, two Akt-inhibitor-sensitive cell lines examined (BT-474 and CAMA-1) and one cell line displaying high sensitivity to MK-2206 and intermediate sensitivity towards AZD5363 (SUM-52-PE) despite possessing low SGK1 mRNA/protein displayed significant phosphorylation of NDRG1 (Figure 2B). On a long exposure, several other of the Akt-inhibitor-sensitive cells such as T47D also displayed observable phosphorylation of NDRG1 (Figure 2B and Supplementary Figure S4 at http://www.biochemj.org/bj/452/bj4520499add.htm). One possibility to account for this observation would be if NDRG1 were phosphorylated by Akt rather than by SGK in the Akt-inhibitor-sensitive cells. To test this, we treated Akt-inhibitor-sensitive BT-474 (Figure 6A), CAMA-1 (Figure 6B) and T47D (Supplementary Figure S4) cells with increasing doses of either the MK-2206 or AZD5363 Akt inhibitors and strikingly found that both compounds suppressed phosphorylation of NDRG1 similarly to PRAS40. In contrast, neither MK-2206 nor AZD5363 Akt inhibitors, even at high concentrations (1 μM), had any affect on NDRG1 phosphorylation in the Akt-inhibitor-resistant BT-549 (Figure 6C) or MDA-MB-436 (Figure 6D) cells, under conditions in which PRAS40 phosphorylation was inhibited.

### Akt-inhibitor-resistant cells are sensitive to mTOR inhibitors

**Figure 7:**
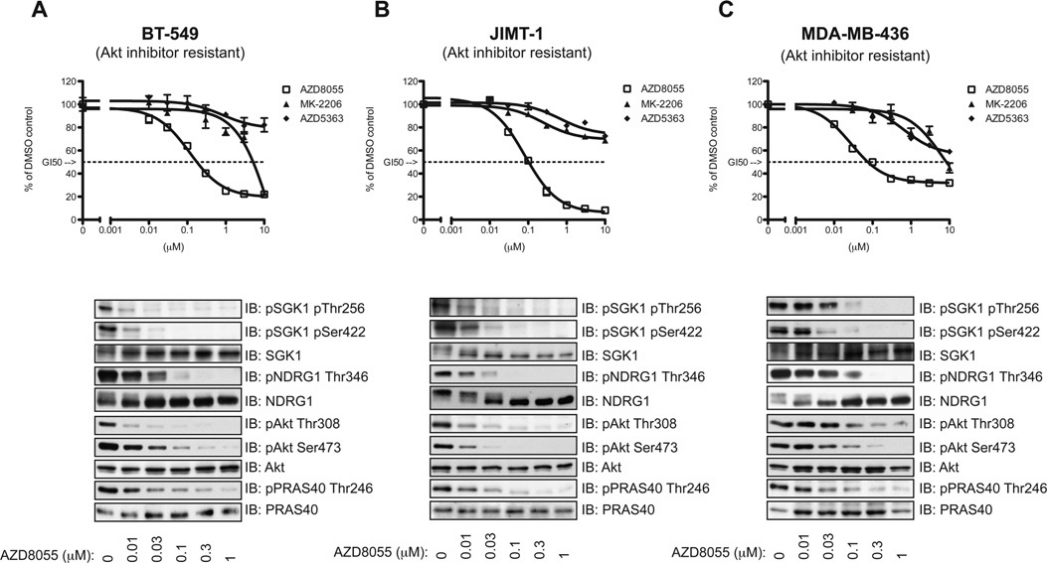
Akt-inhibitor-resistant cells are sensitive to mTOR inhibitors The indicated Akt-inhibitor-resistant cells were exposed to increasing concentrations of the Akt inhibitors (MK-2206 or AZD5363) or an mTOR inhibitor (AZD8055) and 72 h post-inhibitor treatment, cell viability was determined using the MTS assay. Data were fitted to sigmoidal dose–response curves using GraphPad Prism 5.0. The results are the means±S.D. of a triplicate assay and are normalized to the DMSO control that was set to 100%. In the lower panels, cells were treated with the indicated doses of the AZD8055 mTOR inhibitor for 1 h. Cells were lysed and analysed by immunoblotting (IB) with the indicated antibodies. Similar results were observed in two independent experiments. p, phospho-.

As mTOR is a critical activator of SGK1, we investigated whether proliferation of Akt-inhibitor-resistant cells would be sensitive to mTOR inhibitors. This was indeed the case, as proliferation of BT-549 (Figure 7A), JIMT-1 (Figure 7B) and MDA-MB-436 (Figure 7C) cells was suppressed by the AZD8055 mTOR inhibitor [31] (GI_50_ values of ~0.2 μM for BT-549, ~0.1 μM for JIMT-1 and ~0.2 μM for MDA-MB-436). Moreover, AZD8055 also suppressed phosphorylation of the T-loop (Thr^256^) and hydrophobic motif (Ser^422^) of endogenous SGK1 and phosphorylation of NDRG1 in all of the three Akt-inhibitor-resistant cells tested (Figure 7).

## DISCUSSION

Our main conclusion from the research undertaken in the present study is that elevated SGK1 might be deployed to predict resistance of breast-cancer-derived cells to Akt inhibitors. This finding will be of relevance to the numerous clinical trials evaluating the therapeutic potential of Akt inhibitors for the treatment of cancer. In future work it would be important to gauge whether the cancers most responsive to Akt inhibitors do indeed possess low levels of SGK1 protein/mRNA. The present study also emphasizes that caution is required when employing NDRG1 as a surrogate marker for SGK1 activity. We observe in several breast cancer cells displaying high Akt activity and low SGK1 (including BT-474, CAMA-1 and T47D) that NDRG1 is still phosphorylated and that NDRG1 phosphorylation is suppressed by Akt inhibitors. This indicates that, at least in these cancer cells, Akt, rather than SGK1, is phosphorylating NDRG1. Previous studies have shown that Akt can phosphorylate NDRG1 *in vitro*, albeit less efficiently than SGK1 [25]. In contrast, in the cancer cell lines displaying high levels of SGK1, we find that NDRG1 phosphorylation is insensitive to Akt inhibitors and knockdown of SGK1 inhibits NDRG1 phosphorylation. On the basis of these observations, we recommend that in future clinical studies, in addition to examining SGK1 protein/mRNA expression, it would be important, if feasible, to monitor the effect that administration of Akt inhibitors has on NDRG1 phosphorylation. Finding that an Akt inhibitor robustly suppresses NDRG1 phosphorylation would indicate that the tumour has high Akt, but low SGK1, activity. Our prediction would be that these tumours would be more sensitive to Akt inhibitors. In contrast, if administration of an Akt inhibitor failed to suppress NDRG1 phosphorylation, this would be a sign that SGK1 activity was elevated, and that the tumours would be likely to be resistant to Akt inhibitors. Akt-inhibitor-resistant tumours displaying elevated SGK1 might be better treated with mTOR inhibitors that suppress SGK1 activity.

SGK1 has been reported to phosphorylate NDRG1 at three C-terminal residues (Thr^328^, Ser^330^ and Thr^346^) and NDRG2 in the corresponding residues [25]. Phosphorylation of NDRG1 by SGK1 primes NDRG1 for further phosphorylation by GSK3 at another three residues (Ser^342^, Ser^352^ and Ser^362^) [25]. The precise molecular function of NDRG1 is unknown and consequently the role of its phosphorylation by SGK1/Akt and GSK3 remains uncharacterized. NDRG1 expression is regulated via multiple mechanisms, including up-regulation by stress signals, such as changes to redox potential [32], nickel toxicity [33], DNA damage, elevated p53 [34] and hypoxia [35], and down-regulation by the proto-oncogene N-Myc [36]. Both oncogenic and tumour suppressive roles have been proposed for NDRG1. Although decreased NDRG1 expression has been described in a number of tumour types, including breast cancer, increased NDRG1 expression has also been described in a number of cancers [37]. It is unclear whether these contrasting observations could be due to tissue-specific functions of NDRG1. Several studies have correlated the levels of NDRG1 expression with invasiveness and proliferation [37]. For example, ectopic overexpression of NDRG1 in MDA-MB-468 breast cancer cells is reported to suppress *in vitro* invasiveness [38] and ectopic overexpression of NDRG1 in cultured MCF-7 breast cancer cells is reported to suppress proliferation rate [34]. The effect of SGK1 knockdown on reducing the proliferation rate of Akt-inhibitor-resistant cell lines and the invasive ability of BT-549 cells could therefore be at least partially mediated via altered function of NDRG1 due to its dephosphorylation. In future it would be of interest to dissect the precise molecular role(s) that phosphorylation of NDRG1 by SGK1/Akt and GSK3 plays.

SGK1 expression is also markedly induced by many steroid hormones, including the glucocorticoid dexamethasone [39], that are routinely used to reduce swelling in cancer patients. This raises the possibility that administration of steroid hormones to cancer patients receiving Akt inhibitors might have the potential to induce SGK1 in tumour cells and thereby induce resistance to Akt inhibitors. Previous work has shown that treatment of cancer cell lines with dexamethasone promotes cell survival, an effect that is counteracted by knockdown of SGK1 [40]. This also emphasizes the key role that SGK1 activity can play in driving the expansion of tumour cells. Indeed, by promoting induction of SGK1, steroid treatment could have the potential to promote proliferation of all cancers.

Our results also demonstrate that, in the four Akt-inhibitor-resistant breast cancer cell lines displaying elevated SGK1 evaluated (BT-549, JIMT-1, MDA-MB-436 and HCC-1937), knockdown of SGK1 markedly suppressed cell proliferation. This effect was rescued by re-expression of wild-type, but not kinase-inactive, SGK1. Knockdown of SGK1 did not diminish Akt phosphorylation or phosphorylation of the Akt substrate PRAS40 (Supplementary Figure S5 at http://www.biochemj.org/bj/452/bj4520499add.htm), indicating that SGK1 can promote proliferation and survival of these cells independently of Akt. In future studies it would be important to establish whether potent and selective inhibitors of SGK1 would inhibit proliferation of tumour cells displaying elevated SGK1 activity. Moreover, as Akt and SGK1 are related protein kinases, it might be feasible to develop inhibitors that target both enzymes. It would be fascinating to evaluate the efficacy of a dual Akt/SGK inhibitor at suppressing growth of cancer cells exhibiting elevated SGK1 activity. It should be noted that mechanisms other than enhanced SGK1 activity are also likely to contribute to the resistance to Akt inhibitors. Indeed, one of the Akt-inhibitor-resistant breast cancer cell lines we have analysed (MDA-MB-157) displays low SGK1 levels and NDRG1 phosphorylation (Figure 2B). This emphasizes the importance of future work to profile a much larger number of breast and other types of cancer cells to establish the proportion of different tumours that are resistant to Akt inhibitors and also display elevated SGK1 as well as elevated NDRG1 phosphorylation that is not suppressed by Akt inhibitors.

In conclusion, this is the first study to report on the signalling pathways that mediate innate resistance of breast cancer cells to Akt inhibitors. Our findings suggest that elevation of SGK1 expression represents one mechanism predicting Akt inhibitor resistance. We advocate that monitoring NDRG1 phosphorylation responses following administration of Akt inhibitors could represent an effective general biomarker to assess SGK1 activity in tumour cells. Our findings indicate that the breast cancers most likely to be sensitive to Akt inhibitors would be those displaying high Akt, low SGK1 mRNA/protein and in which phosphorylation of NDRG1 is suppressed by Akt inhibitors. In contrast, tumours displaying elevated SGK1 mRNA/protein in which NDRG1 phosphorylation is not suppressed by Akt inhibitors are likely to be more resistant to Akt inhibitors. Such tumours might be better treated with signal transduction suppressors that reduce SGK1 activity, such as mTOR inhibitors. We also feel that more work is needed to determine whether administration of steroids to patients has the potential to induce SGK1 expression and cause resistance to Akt inhibitors. Finally, it would be of immense interest to explore the therapeutic utility of SGK1 inhibitors or dual Akt/SGK1 inhibitors in treating Akt-resistant cancer cells possessing elevated SGK1.

## Online data

Supplementary data
