# Supplementary material for: Elevated SGK1 predicts resistance of breast cancer cells to Akt inhibitors
Source: Biochem J. 2013 May 31;452(Pt 3):499–508. doi: 10.1042/BJ20130342 (PMC3671793; doi:10.1042/BJ20130342)
Supplement: Supplementary data [file bj4520499add.pdf]

BJ ChemBio

Biochem. J. (2013) **452**, 499–508 (Printed in Great Britain) doi:10.1042/BJ20130342

online data online commentary

SUPPLEMENTARY ONLINE DATA

Elevated SGK1 predicts resistance of breast cancer cells to Akt inhibitors

Eeva M. SOMMER*1, Hannah DRY†, Darren CROSS†, Sylvie GUICHARD†, Barry R. DAVIES† and Dario R. ALESSI*1

*MRC Protein Phosphorylation and Ubiquitylation Unit, College of Life Sciences, University of Dundee, Dundee DD1 5EH, U.K., and †Oncology iMED, AstraZeneca, Alderley Park, Cheshire SK10 4TG, U.K.

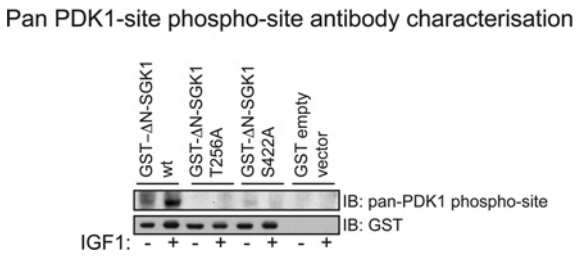

Figure S1 Characterization of pan-PDK1-site antibody (CST #9379) for monitoring SGK1 Thr^{256} phosphorylation

HEK-293 cells were transiently transfected with either wild-type, T256A or S422A GST (glutathione transferase)–ΔN-SGK1. At 24 h post-transfection, cells were serum starved for 16 h, stimulated with 50 ng/ml IGF1 (insulin-like growth factor 1) for 30 min, lysed and subjected to immunoblot (IB) analysis with the indicated antibodies. Mutation of the hydrophobic motif Ser^{422} to an alanine residue prevents PDK1 from phosphorylating the T-loop (Thr^{256}) of SGK1, as mTORC2 phosphorylates the hydrophobic motif of SGK1 creating a docking site for PDK1 that subsequently phosphorylates Thr^{256} [1].

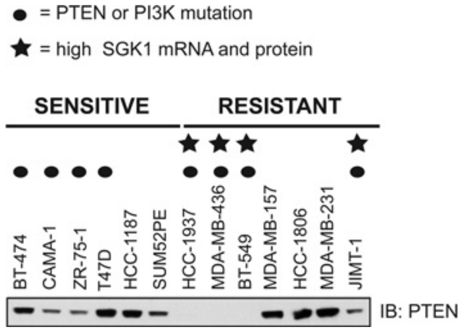

Figure S2 PTEN protein expression in the cell lines used

Total protein was isolated from the cells indicated and the lysates were analysed by immunoblotting (IB) with anti-PTEN antibody.

[1] Correspondence may be addressed to either of these authors (email e.sommer@dundee.ac.uk or d.r.alessi@dundee.ac.uk).

© 2013 The Author(s)
The author(s) has paid for this article to be freely available under the terms of the Creative Commons Attribution Licence (CC-BY) (http://creativecommons.org/licenses/by/3.0/) which permits unrestricted use, distribution and reproduction in any medium, provided the original work is properly cited.

Biochemical Journal www.biochemj.org

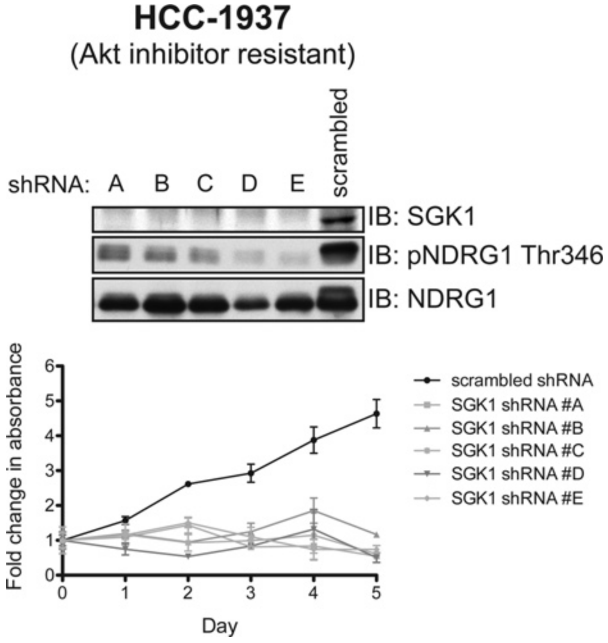

Figure S3 SGK1 knockdown reduces NDRG1 phosphorylation and suppresses proliferation in HCC-1937 cells

HCC-1937 cells were transduced with lentiviral SGK1 and scrambled shRNAs. For experiments shown in the upper panel, cells were lysed 72 h post-infection and the lysates were analysed with the indicated antibodies by immunoblotting (IB). For the experiments shown in the lower panel, equal numbers of cells were seeded 48 h post-infection on to 96-well plates and allowed to adhere overnight. Cell proliferation was then determined over a 5 day period by carrying out the MTS assay at 24 h intervals. Each data point is the average MTS assay of samples assayed in triplicate ± S.D. Data are presented as fold change relative to day 0 values (day 0 equals 24 h post-seeding). Similar results were observed in at least two independent experiments. p, phospho-.

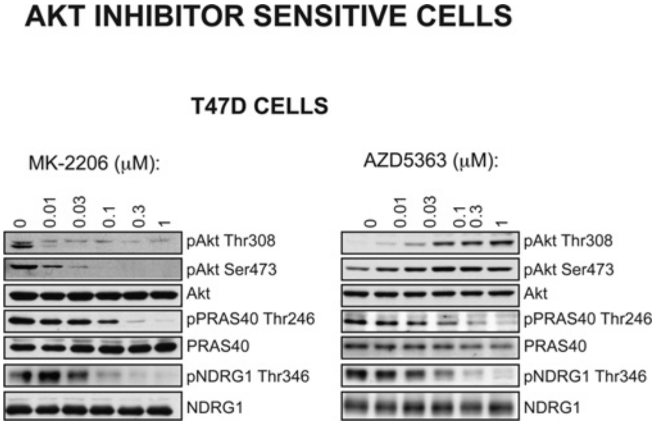

Figure S4 Evidence that Akt mediates phosphorylation of NDRG1 in Akt-inhibitor-sensitive cells

T47D cells were treated with the indicated doses of the MK-2206 or AZD5363 Akt inhibitors for 1 h. Cells were lysed and analysed by immunoblotting with the indicated antibodies. Similar results were observed in two independent experiments.

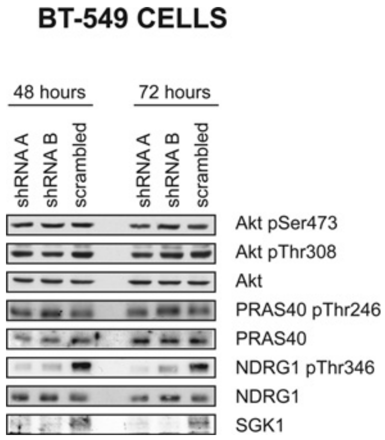

Figure S5 Knockdown of SGK1 does not affect Akt signalling

BT-549 cells were transduced with lentiviral SGK1 and scrambled shRNAs. Cells were lysed at 48 and 72 h post-infection and the lysates were analysed with the indicated antibodies by immunoblotting.

Table S1 Sequences and TRC numbers of the SGK1 shRNAs used in the present study

shRNA	TRC number	Sequence (5′→3′)
A	TRCN0000040175	CCGGCGGAATGTTCTGTTGAAGAATCTCGAGATTCTTCAACAGAACATTCCGTTTTTG
B	TRCN0000040177	CCGGCATGTCTTCTTCTCCTTAATTCTCGAGAATTAAGGAGAAGAAGACATGTTTTTG
C	TRCN0000010432	CCGGCAATTCTCATCGCTTTCATGACTCGAGTCATGAAAGCGATGAGAATTGTTTTTG
D	TRCN0000196562	CCGGGCAATCTTATTGCACACTGTTCTCGAGAACAGTGTGCAATAAGATTGCTTTTTTG
E	TRCN0000194957	CCGGCTGGAAGCTTAGCAATCTTATCTCGAGATAAGATTGCTAAGCTTCCAGTTTTTTG
Scrambled		CCTAAGGTTAAGTCGCCCTCGCTCTAGCGAGGGCGACTTAACCTTAGG

© 2013 The Author(s)
The author(s) has paid for this article to be freely available under the terms of the Creative Commons Attribution Licence (CC-BY) (http://creativecommons.org/licenses/by/3.0/) which permits unrestricted use, distribution and reproduction in any medium, provided the original work is properly cited.

Table S2 Sequences of primers used for qRT-PCR in the present study

Primer name	Sequence (5′→3′)
Human SGK1 sense	CATAGGAGTTATTGGCAAT
Human SGK1 antisense	CTTCCATCTCACTAACCA
Human SGK2 sense	CTTTGTTATTAGGGATAGTCA
Human SGK2 antisense	GAAGTGAATGGTTTGTCT
Human SGK3 sense	ATATTCTCTTGGCAGGAA
Human SGK3 antisense	AATGGCTCATTAAATCAGTT
Human 18S sense	CTAGAATTACCACAGTTATCC
Human 18S antisense	CTAGAATTACCACAGTTATCC

Table S3 Summary of known mutations in the 13 breast cancer cell lines used in the present study

The majority of the mutation information was obtained from the Cosmic database [2] available online at http://cancer.sanger.ac.uk/cancergenome/projects/cosmic/.

Cell line	Gene mutated or amplified	Mutation (amino acid)	Reference
BT-474	*PIK3CA*	K111N	Cosmic [2]
	P53	E285K	Cosmic [2]
CAMA-1	*CDH1*	?	Cosmic [2]
	PTEN	Thr^{277} frameshift	Cosmic [2]
	PTEN	D29H	Cosmic [2]
	P53	R280T	Cosmic [2]
ZR-75-1	*PTEN*	L108R	Cosmic [2]
T47D	*PI3KCA*	H1047R	Cosmic [2]
	P53	L194F	Cosmic [2]
HCC-1187	*P53*	Gly^{108} deletion	Cosmic [2]
SUM52PE	*CDKN2A*	A68V	Cosmic [2]
	FGFR2	Overexpression	[3]
HCC-1937	*BRCA1*	Gln^{1756} frameshift	Cosmic [2]
	P53	Arg^{306} stop	Cosmic [2]
	PTEN	Deletion of exons 1–9	[4]
MDA-MD-436	*BRCA1*	?	Cosmic [2]
	RB1	Gly^{203} frameshift	Cosmic [2]
BT-549	*PTEN*	Val^{275} frameshift	Cosmic [2]
	RB1	?	Cosmic [2]
	P53	R249S	Cosmic [2]
MDA-MB-157	*NF1*	Ser^{275} frameshift	Cosmic [2]
	P53	Ala^{88} frameshift	Cosmic [2]
	MSH6	R644S and P42S	Cosmic [2]
HCC-1806	*LKB1*	Deletion	Cosmic [2]
	P53	Thr^{256} frameshift	Cosmic [2]
	KDM6A	Deletion	Cosmic [2]
	CDKN2a(*p14*)	Deletion	Cosmic [2]
	CDKN2A	Deletion	Cosmic [2]
MDA-MB-231	*BRAF*	G464V	Cosmic [2]
	CDKN2A	Deletion	Cosmic [2]
	CDKN2a(*p14*)	Deletion	Cosmic [2]
	KRAS	G13D	Cosmic [2]
	NF2	Glu^{231} stop	Cosmic [2]
	P53	R280K	Cosmic [2]
JIMT-1	*HER-2*	Amplification	[5]
	PIK3CA	C420R (T1258C)	[6]

REFERENCES

1 García-Martínez, J. M. and Alessi, D. R. (2008) mTOR complex 2 (mTORC2) controls hydrophobic motif phosphorylation and activation of serum- and glucocorticoid-induced protein kinase 1 (SGK1). Biochem. J. **416**, 375–385

2 Forbes, S. A., Bindal, N., Bamford, S., Cole, C., Kok, C. Y., Beare, D., Jia, M., Shepherd, R., Leung, K., Menzies, A. et al. (2011) COSMIC: mining complete cancer genomes in the Catalogue of Somatic Mutations in Cancer. Nucleic Acids Res. **39**, D945–D950

3 Tannheimer, S. L., Rehemtulla, A. and Ethier, S. P. (2000) Characterization of fibroblast growth factor receptor 2 overexpression in the human breast cancer cell line SUM-52PE. Breast Cancer Res. **2**, 311–320

4 Hollestelle, A., Elstrodt, F., Nagel, J. H., Kallemeijn, W. W. and Schutte, M. (2007) Phosphatidylinositol-3-OH kinase or RAS pathway mutations in human breast cancer cell lines. Mol. Cancer Res. **5**, 195–201

5 Tanner, M., Kapanen, A. I., Junttila, T., Raheem, O., Grenman, S., Elo, J., Elenius, K. and Isola, J. (2004) Characterization of a novel cell line established from a patient with herceptin-resistant breast cancer. Mol. Cancer Ther. **3**, 1585–1592

6 Koninki, K., Barok, M., Tanner, M., Staff, S., Pitkanen, J., Hemmila, P., Ilvesaro, J. and Isola, J. (2010) Multiple molecular mechanisms underlying trastuzumab and lapatinib resistance in JIMT-1 breast cancer cells. Cancer Lett. **294**, 211–219

Received 4 March 2013/11 April 2013; accepted 15 April 2013
Published as BJ Immediate Publication 15 April 2013, doi:10.1042/BJ20130342

© 2013 The Author(s)
The author(s) has paid for this article to be freely available under the terms of the Creative Commons Attribution Licence (CC-BY) (http://creativecommons.org/licenses/by/3.0/) which permits unrestricted use, distribution and reproduction in any medium, provided the original work is properly cited.
